# Designer patterned functional fibers via direct imprinting in thermal drawing

**DOI:** 10.1038/s41467-020-17674-8

**Published:** 2020-07-31

**Authors:** Zhe Wang, Tingting Wu, Zhixun Wang, Ting Zhang, Mengxiao Chen, Jing Zhang, Lin Liu, Miao Qi, Qichong Zhang, Jiao Yang, Wei Liu, Haisheng Chen, Yu Luo, Lei Wei

**Affiliations:** 1 School of Electrical and Electronic Engineering, Nanyang Technological University, 50 Nanyang Avenue, Singapore, 639798 Singapore; 2 Institute of Engineering Thermophysics, Chinese Academy of Sciences, Beijing, 100190 China; 3 CNRS/NTU/THALES, UMI3288, Research Techno Plaza, 50 Nanyang Drive, Singapore, 637553 Singapore

**Keywords:** Surface patterning, Nanophotonics and plasmonics, Sensors, Design, synthesis and processing

## Abstract

Creating micro/nanostructures on fibers is beneficial for extending the application range of fiber-based devices. To achieve this using thermal fiber drawing is particularly important for the mass production of longitudinally uniform fibers up to tens of kilometers. However, the current thermal fiber drawing technique can only fabricate one-directional micro/nano-grooves longitudinally due to structure elongation and polymer reflow. Here, we develop a direct imprinting thermal drawing (DITD) technique to achieve arbitrarily designed surface patterns on entire fiber surfaces with high resolution in all directions. Such a thermal imprinting process is simulated and confirmed experimentally. Key process parameters are further examined, showing a process feature size as small as tens of nanometers. Furthermore, nanopatterns are fabricated on fibers as plasmonic metasurfaces, and double-sided patterned fibers are produced to construct self-powered wearable touch sensing fabric, revealing the bright future of the DITD technology in multifunctional fiber-based devices, wearable electronics, and smart textiles.

## Abstract (web summary)

Creating micro/nanostructures on fibers is beneficial to many fiber-based devices, which remains a challenge in large-scale fabrication due to elongation and reflow. Here, the authors demonstrate a method for generating high-resolution, arbitrarily designed surface patterns on fiber during the thermal drawing process.

## Introduction

Scalable fabrication of flexible fibers with various functions such as sensing^1–3^, actuating^4–6^, imaging^7–9^, energy harvesting^10,11^, and light-emitting^12,13^ is highly demanded to support the rapid development of fiber-based devices and their wide range of applications. A typical top-down approach, thermal fiber drawing, has been broadly employed for the mass production of longitudinally uniform fibers with the extended length up to tens of kilometers^14^. The thermal fiber drawing process begins with heating a macroscopic preform which mainly consists of glassy material such as a thermoplastic material above its glass transition temperature till soft. The soft preform with a typical diameter in centimeter range is then pulled under a controlled speed to form viscous flow^15^ and thus elongated into fiber with a uniform diameter ranging from tens of micrometers to several millimeters. As the cross-sectional architecture of the fiber is consistent with the preform except for being scaled-down in size after the drawing process, fiber with complex inner structure could be achieved simply by constructing the corresponding inner structure in the macroscopic preform^16^. Besides, this thermal fiber drawing process is applicable to a wide selection of materials including metals^17^, semiconductors^18^, and even liquids^19^ that could be incorporated into fibers under good confinement of the glassy materials thanks to their high viscosity (typically 10^4^–10^8^ Pa·s) during the thermal fiber drawing process. Combining the designed inner structures with different functional materials, the recent studies have proposed numerous functional fibers applied in optoelectronics where an innovative laser-based approach was developed for controlling crystal nucleation and growth leading to high-performance photodetectors^20^, motion sensing via a simple contact resistance mechanism^21,22^, thermal sensing based on thermoelectric effects^23,24^, energy transducing via piezoelectric effects^4,25^ and bio-fiber interfaces achieved through miniaturized probes incorporating multiple functions^26,27^.

Beyond focusing on fiber’s material selections and inner structures, creating micro/nanostructures on fiber surface will enable many unique properties onto the current functional fibers to extend their application fields, such as hydrophobic surface, coloration, enhanced gas absorption, tunable plasmonic behavior, increased surface area, and antimicrobial. However, by far only surface grooves along the fiber’s axial direction were achieved^15,28,29^. To create high-resolution complex surface patterns in arbitrary directions, two fundamental challenges must be addressed. The first challenge is the structure elongation when a preform is drawn into a fiber. Typically, the resulting fiber is 2–6 orders of magnitude longer than the original preform. Although a well-controlled elongation ensures the uniformity along the entire fiber, it in turn stretches all the pre-defined structures on the preform by the same orders of magnitude along the axial direction. This is the main reason that all the reported surface patterns to date are aligned along the axial direction. The other challenge is the reflow of heated thermoplastic materials driven by surface tension^15^, which leads to the distortion of the desired structures, thus resulting in low pattern resolution. Sorin et al. have studied the reflow behavior and improved the pattern resolution to sub-micrometer by introducing a polymer interface with low surface tension^28^. However, choosing two different polymers to construct low surface tension interface not only largely restricts the selection of applicable materials, but also reduces the compatibility with other inner functional structures, which essentially obstructs the further development and applications of surface patterned fibers. Therefore, an effective yet universal approach to creating high-resolution complex micro/nanostructure beyond axial direction on thermally drawn fibers is vastly demanded.

In this work, we propose and demonstrate a direct imprinting in thermal drawing (DITD) technique to address the above mentioned challenges and achieve high-resolution designer micro/nanostructures on fiber surface with good compatibility on both materials and inner structures. This technique is based on the fact that the thermoplastic materials can be re-shaped during the thermal drawing process by directly imprinting high-resolution patterns in arbitrary directions on the soft fiber surface after the elongation^30–33^, while the imprinted fiber can be cooled down rapidly to significantly restrain reflow. Using this technique, a wide variety of regular and irregular surface patterns are created on hundreds-meter long fibers with different materials and inner structures, illustrating the high stability, high yield, and good compatibility of the DITD technique. To understand this process, temperature distribution during the DITD process is studied by simulation and verified by experimental results. Moreover, key process parameters such as resolution, depth control, and repeatability are examined, exhibiting the feature size as small as tens of nanometers. Plasmonic behaviors of nanopatterned fibers are investigated to demonstrate their potential in optical applications. Furthermore, to reveal this technique’s extensive application prospect and good compatibility with functional fibers, triboelectric nanogenerators (TENGs) are fabricated based on fibers with both flat and patterned surfaces. The output signals show that the surface pattern can significantly enhance TENG’s performance. Finally, a self-powered wearable multipoint touch sensing fabric is successfully built based on the patterned TENG fibers, indicating the DITD technique’s promising future in wearable electronics and smart textiles^34–37^.

## Results

### Creating surface patterns on fibers with different materials

**Fig. 1:**
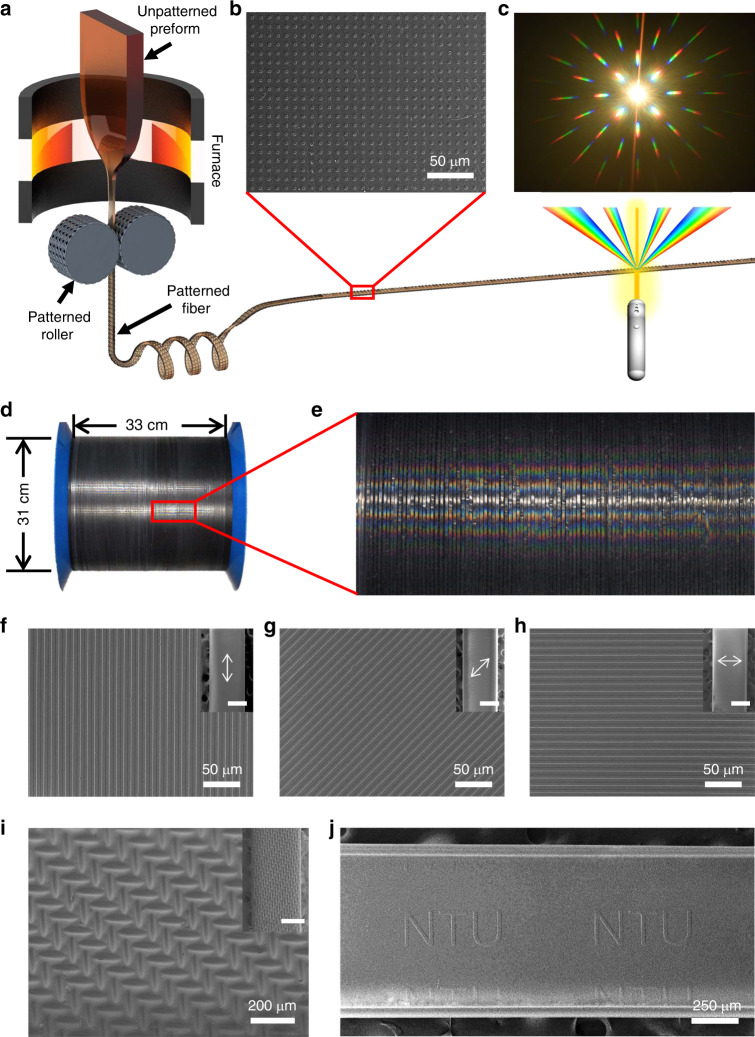
Fabrication of various surface patterns via direct imprinting in thermal drawing (DITD) technique. **a** Schematic of the DITD process for producing 2D diffraction grating on fiber surface. **b** SEM image of 2D microdot array on fiber surface. **c** Diffraction patterns when illuminated by a white supercontinuum laser. **d** 300-meter-long fully patterned fiber collected on a cylindrical bobbin. **e** Enlarged photograph showing the diffraction pattern on fiber-wrapped bobbin. **f**–**h** Parallel lines with both linewidth and spacing of 5 μm created in different directions (parallel, perpendicular, and 45° angle to the fiber’s axial direction). Inset: overview of patterned fibers. The double arrows represent the groove directions (scale bar, 500 μm). **i** Surface pattern with nonuniform depth. Inset scale bar, 500 μm. **j** Fiber patterned with customized letters “NTU”.

Compared to the traditional thermal fiber drawing process, the DITD process introduces a pair of rollers with desired surface structures as templates to thermally imprint surface patterns onto the drawn fiber. As sketched in Fig. 1a, the DITD process starts from feeding a preform into furnace slowly to heat it above glass transition temperature till soft. Then a neck-down region is formed in the heating zone as the softened preform is scaled down cross-sectionally and elongated into fiber under a constant downward drawing speed. A pair of patterned rollers are fixed at both sides of the fiber right below the neck-down region. Since the temperature of the neck-down region is still high enough to reshape the fiber, an exactly inverted surface pattern can be created on both sides of the drawn fiber after being directly pressed by the patterned rollers acting as templates. Meanwhile, the patterned fiber cools down rapidly because of the physical contact with the rollers, which greatly restricts subsequent distortion caused by reflow^15^. While the fiber is being drawn down continuously, the rollers keep rotating to imprint patterns onto fiber surfaces smoothly and repeatedly, creating a continuous surface pattern on the entire length of drawn fiber via the DITD process. A demonstration of this continuous fabrication process is shown in Supplementary Movie 1.

The surface pattern of rollers can be fabricated either by directly modifying the roller surface or simply attaching a patterned flexible substrate on the roller surface. Therefore, almost all of the processes for fabricating micro/nanostructure such as self-assembly^38^, lithography^39,40^, focused ion beam (FIB) etching^41^, and laser machining^42,43^ could be used for preparing the patterned rollers, which enables the DITD technique to fabricate fibers with a wide variety of surface patterns. In our initial experiment, a polyethylene terephthalate (PET)-based two-dimensional (2D) micro-hole array was attached on both rollers while an unpatterned polyvinylidene fluoride (PVDF) bar was chosen as the preform (see Methods for the detailed information). As a result, PVDF fiber with the inverted surface pattern, 2D microdot array, on both sides was successfully fabricated via the DITD technique, which is verified by scanning electron microscope (SEM) image in Fig. 1b. This surface pattern was further tested under illumination by white supercontinuum laser as the 2D microdot array served as a 2D diffraction grating. After the white supercontinuum laser propagated through the patterned PVDF fiber, the laser was split into several beams in the spatial domain and laser with different wavelengths was also separated in different diffraction angles in the presence of the 2D diffraction grating. Thus, a sharp 2D diffraction pattern with “rainbow” colors was formed and captured in Fig. 1c, proving the high quality of the periodic surface pattern on PVDF fiber. It is worth mentioning that the DITD technique is a high throughput process with excellent reliability to produce a single continuous patterned fiber. As exhibited in Fig. 1d, e, a 300-meter-long fiber with 2D dot array fully covered on both sides was fabricated and collected on a capstan. The diffraction pattern can be observed on the whole capstan, indicating that the surface pattern is created continuously on the entire surface of the 300-meter-long fiber.

Next, a series of fibers with various surface patterns were fabricated under the same conditions using different templates attached on the rollers. Firstly, parallel lines with both width and spacing of 5 μm in three directions (parallel, perpendicular, and 45° angle to the fiber’s axial direction) were created on the surface of PVDF fibers, as shown in Fig. 1f–h. The strict periodicity and sharp edges confirmed the high resolution of the surface pattern. Secondly, a more complex pattern (Fig. 1i) with nonuniform depth was fabricated by changing the template to a 1000-mesh stainless steel screen. Insets of Fig. 1f–i present the overview of the respective micropatterned fiber (all the scale bars in the insets are 500 μm). Further, the ability of imprinting in all directions and high resolution enable the DITD technique to produce fibers with arbitrarily designed surface pattern. As displayed in Fig. 1j, customized letters “NTU” was successfully imprinted on the fiber surface.

Knowing that almost all the thermoplastic materials are applicable to the imprinting process, the DITD process is in principle compatible with all the thermoplastic materials employed in thermal fiber drawing process, including amorphous polymers such as polycarbonate (PC), polyethyleneimine (PEI), cyclic olefin copolymer (COC), and polystyrene (PS), semicrystalline polymers such as polyether ether ketone (PEEK) and PVDF, and elastomers such as Santoprene and styrene-ethylene-butylene-styrene (SEBS). As a verification, fibers drawn from PC, PEI, PEEK, and SEBS with the same surface pattern were fabricated through the DITD process (Supplementary Fig. 6). Furthermore, the surface pattern does not influence the instinct properties of the materials. As shown in Supplementary Fig. 7, SEBS fiber with surface pattern preserved its superior flexibility as it was stretched to 600% of its original length without breaking. The high compatibility on materials selection offers the DITD technique great potentials in a wide range of applications.

### Thermal behavior study of DITD process

**Fig. 2:**
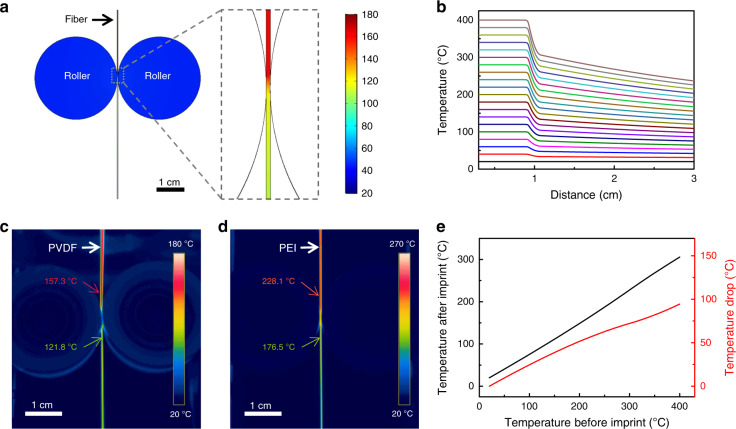
Thermal behavior during the DITD process. **a** Simulation of temperature distribution by setting fiber temperature to 160 °C before contacting rollers. **b** Simulated temperature distribution along fibers under different drawing temperatures. Rollers were placed at 1 cm. **c** Thermal image of PVDF fiber during the DITD process. **d** Thermal image of PEI fiber during the DITD process. **e** Simulated fiber temperature after the imprinting process under different drawing temperatures (black line) and simulated temperature drop under different drawing temperatures (red line).

Studying the thermal behavior of the fiber is crucial to gain a better understanding of the DITD process. Since the thermal process of fiber drawing has been indepthly studied in earlier works^15,28^, we only focus on the thermal process of surface pattern imprinting after the fiber is drawn out of the neck-down region. Finite element analysis was conducted by COMSOL to simulate heat transfer among fiber, rollers, and ambient air as well as thermal radiation of all surfaces. All the geometry and parameters were defined according to the actual sizes and materials used in our experiment. Figure 2a shows the simulated temperature distribution by setting fiber temperature to 160 °C before contacting with the rollers. And the temperature of the fiber drops rapidly to ~118 °C after being pressed by rollers, as shown in the enlarged area of Fig. 2a. The rapid temperature drop during the contact is mainly caused by two factors. One factor is the thermal conductivity contrast between the roller and air. The roller we used is made of steel, whose thermal conductivity coefficient is around 30 W m^−1^ K^−1^, which is about 3 orders of magnitude higher than that of air. Thus, the heat transfer between roller and fiber is much faster than that between roller and air under the same temperature difference. The other factor is the total heat capacity contrast between roller and fiber. During the contact, the fast heat transfer leads to a rapid temperature drop of the fiber because of its low heat capacity, while the temperature of the roller only increases a little because of the high heat capacity, which helps maintain a high temperature difference. Additionally, the large roller size could also help in dissipating heat form roller to air. Thus, the temperature of the roller could be maintained at a relatively low value by absorbing heat from the fiber and dissipating heat to air simultaneously. Considering that the drawing temperature varied with different materials, we further ran a parameter sweep to obtain the temperature distribution along the fiber under different drawing temperatures. To simplify the simulation, we assumed that the temperature of fiber was constant before contacting with rollers. The temperature as a function of the distance from the neck-down region is plotted in Fig. 2b. A rapid temperature drop under all drawing temperatures is observed at the distance of 1 cm, which is the position of the rollers in our geometry model. The higher the drawing temperature used, the more the temperature drops when being pressed by rollers. Additionally, at a larger distance, the temperature keeps decreasing as the fiber leaves rollers and is drawn downward. The smaller curve slops after the fiber left the rollers indicate that the thermal contact between fiber and rollers dominates the heat transfer in the imprinting process. To verify the simulation results, thermal images of PVDF and PEI were captured by thermal camera during the DITD process, as shown in Fig. 2c, d, respectively. The observed temperature distribution in Fig. 2c matches with the simulated results very well. And the temperature distribution of PEI fiber in Fig. 2d follows the similar trend with the simulated temperature distribution shown in Fig. 2b. Also, a temperature drop of 35.5 °C and 51.6 °C is observed after PVDF and PEI fiber is pressed by rollers, respectively.

Based on the simulation and experimental results, we can divide the thermal imprint process into two phases for more detailed discussion. Phase 1 is the surface pattern creating by pressing the template to high-temperature fiber. In this phase, the polymer on fiber surface fills into the cavities on the template to form an inverted pattern under pressure. The polymer flow behavior and important factors affecting the flow behavior are discussed in detail in Supplementary Note 1. As the fiber is drawing down continuously during the DITD process, each point on the fiber surface only contacts with the template for a limited time. Therefore, the filling time must be shorter than the contact time to ensure a high-fidelity pattern. In our case, the contact time is ~50 ms as estimated in Supplementary Note 2, while the filling time can be decided by the following equation^31^:

1 $$t = \frac{{12\eta Sh_{\mathrm{c}}^2}}{{P\left( {2W} \right)^2\left( {S + W} \right)}}$$

Where $\eta$ is the viscosity, *S* and *W* are the half widths of intender and cavity respectively, *h*_*c*_ is the depth of cavity, and *P* is imprinting pressure. Here, we take the template used in Fig. 1f for example, where *S*, *W*, and *h*_c_ are 2.5 × 10^−6^ m, 2.5 × 10^−6^ m, and 1 × 10^−7^ m, respectively. *P* is estimated to be 5.7 MPa as discussed in Supplementary Note 2, while a typical viscosity of 10^6^ Pa·s is used. Thus, the filling time can be estimated to be 0.42 ms, which is much shorter than the contact time of 50 ms. Phase 2 is the cooling down of the patterned fiber when contacting with rollers, which helps hinder reflow deformation after the patterned surface is separated from the templates. Figure 2e shows the simulated temperature drop of the fiber being cooled down by the rollers under different drawing temperatures. We can find that the rollers decrease the fiber’s temperature by more than 35 °C in general as the commonly used materials in fiber drawing are always drawn above 150 °C. For semicrystalline polymers such as PVDF and PEEK, such a significant temperature drop will solidify them immediately as the relatively strong intermolecular forces will prevent them from softening below the melting temperature^44^. Hence, the reflow should be negligible after being cooled down by the rollers. While for most amorphous materials such as PC and PEI, the temperature will directly drop below their glass transition temperature (*T*_g_) after passing through the rollers, thus reflow is also prevented. Only for materials with a relatively low *T*_g_ such as PS, the temperature may stay higher than their *T*_g_ after being separated from rollers. Then, we can estimate the characteristic reflow time for a periodic surface pattern which is defined as $\tau = \lambda \eta /\pi \gamma$^28^, where $\lambda$ is the period and $\gamma$ is surface tension. The viscosity will significantly increase when temperature decreases, e.g., the viscosity of PS will increase by 3 orders of magnitude when the temperature drops from 170 °C to 130 °C^45^. Here we take PS’s viscosity and surface tension as 10^8^ Pa·s and 40 mN/m at 130 °C^46^. In case of imprinting a pattern with a period of 10^−6^ m, the reflow time can be calculated to be 800 s. Noticing that fiber’s temperature will keep decreasing under ambient air after being separated with rollers (Fig. 2b), PS’s temperature will drop below *T*_g_ in few seconds, which is much shorter than the reflow time of 800 s. Combining phase 1 and phase 2, we can conclude that this imprinting process during fiber drawing is in principle compatible with a wide range of materials and patterns. Additionally, we can always adapt the DITD process to a specific material or pattern by adjusting the parameters including temperature, pressure, and drawing speed.

### Key process parameters Investigation of DITD technique

**Fig. 3:**
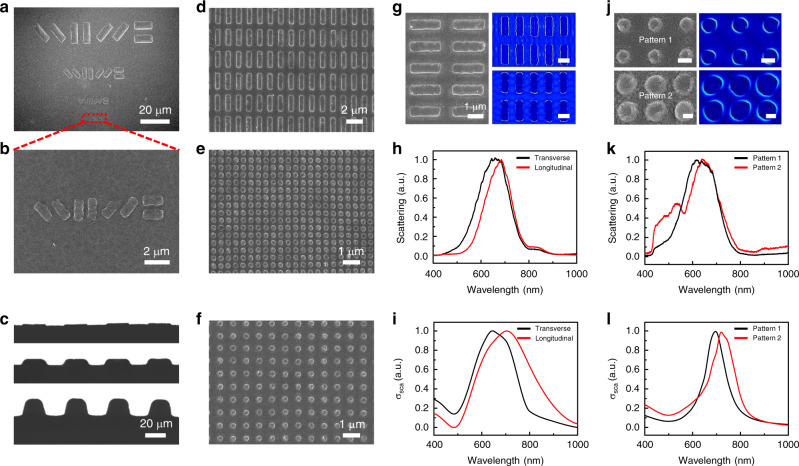
Pattern resolution, depth control, and repeatability tests of the DITD process and plasmonic applications. **a** Resolution test pattern including rectangular blocks with different sizes and orientations fabricated on fiber. **b** Enlarged image showing a feature size in tens of nanometer. **c** Cross-sectional images of the patterned fibers, demonstrating the pattern depths of 2 μm, 8 μm, and 20 μm. **d** Rectangular rod array fabricated on fiber surface. The width and length of the rectangular rods are 700 nm and 2.1 μm. **e** Cylinder array on fiber surface with radius and period of 150 nm and 400 nm. **f** Cylinder array on fiber surface with radius and period of 150 nm and 800 nm. **g** SEM image of the gold nanorods with an aspect ratio of 3 (left) and the corresponding local electric field spatial distributions with transverse (top right, scale bar, 1 μm) and longitudinal laser polarizations (bottom right, scale bar, 1 μm). **j** SEM image and the corresponding local electric field spatial distributions of the gold cylinders with an array period of pattern 1 ≈ 800 nm and pattern 2 ≈ 400 nm. Scale bars, 300 nm for pattern 1 and 150 nm for pattern 2. Dark-field scattering spectra of the gold nanorods (**h**) and gold cylinders (**k**). Simulated scattering cross-section of the gold nanorods (**i**) and gold cylinders (**l**).

A resolution test pattern was designed to find the feature size of the DITD technique. The high-resolution template (Supplementary Fig. 8a) was etched by FIB and an inverted pattern was created on the PVDF fiber surface, as shown in Fig. 3a, b. A series of raised rectangular blocks with different sizes and orientations were created. As demonstrated in the enlarged SEM image, the rectangular blocks with a length of 1.5 μm and a width of 500 nm basically maintained their shapes in all directions except for some distortions on edges, confirming a feature size in tens of nanometers. In view of the slight distortion in the template itself (Supplementary Fig. 8b), the actual resolution of the patterns should be better than it appears. Not only the pattern size, the depth of patterns is also controllable. A set of similarly patterned fibers with depths of 2 μm, 8 μm, and 20 μm were fabricated, as the fibers’ cross-section images show in Fig. 3c. Furthermore, various nanopattern arrays were created on fibers to examine the stability and repeatability of the DITD technique, as presented in Fig. 3d–f. Each array included a large amount of rectangular or circular nanoelements with a predesigned period. The rectangle element was 2.1 μm in length and 700 nm in width, while the round one had a radius of 150 nm. All these nanoelements exhibited high fidelity compared with their templates displayed in Supplementary Fig. 9. The high resolution, controllable depth, and high stability of the DITD technique offer surface patterned fibers extensive application areas.

### Plasmonic behavior of nanopatterned fiber

The ability to combine patterned fiber with plasmonics provides opportunities for a variety of applications of fibers, such as biosensing^47^, bioimaging^48^, and mode converters^49^. Here, we investigated the plasmonic behavior of the nanopatterned fibers (with a 30-nm-thick gold film deposited on top) using dark-field spectroscopy. Finite-difference time-domain (FDTD) simulations were performed and the local electric field spatial distributions of the fiber plasmonic systems are shown in Fig. 3g, j which suggest that hot spots were stimulated. The gold pattern models in FDTD were generated from the SEM images as shown in Fig. 3g left and Fig. 3j left. For the nanorod structures indicated in Fig. 3g, the measured dark-field scattering spectra of the plasmonic fiber with transverse (black line) and longitudinal (red line) laser polarizations are presented in Fig. 3h. Two different resonances correspond to the transverse and longitudinal modes of the nanorods. For the nanocylinder arrays indicated in Fig. 3j (pattern 1 and pattern 2), the dark-field scattering spectra with an incidence of non-polarized light are presented in Fig. 3k. Arrays with a smaller period (see Fig. 3j bottom right) possess longer plasmonic resonance which is induced by the near-field coupling. An additional peak near 520 nm is introduced by the edge roughness. The resulting scattering spectra were reproduced by the simulated scattering cross-sections, as presented in Fig. 3i, l. The slight shift of the resonance peaks for both etched patterns when compared with the experimental characterizations can be attributed to the gold layer surface roughness and the minor edge defects, but a good agreement between the shapes of the measured scatterings and the simulated scattering cross-sections was obtained for different patterns.

### Patterned functional fiber-based TENG

**Fig. 4:**
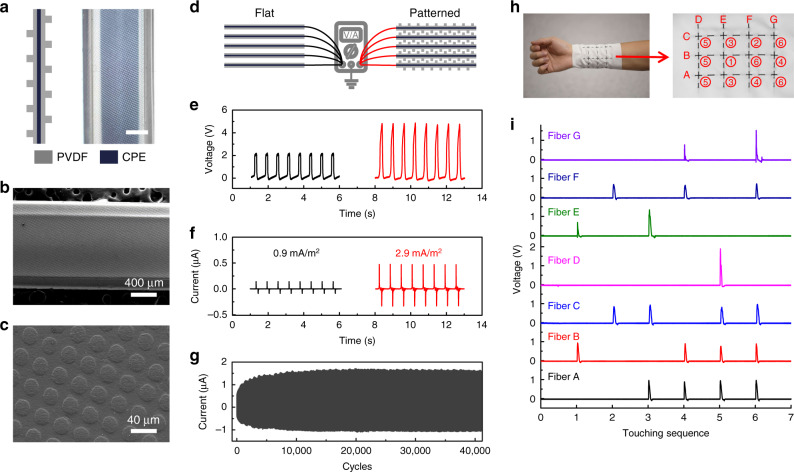
Patterned functional fibers applied in TENG and self-powered wearable multipoint touch sensors. **a** Sketch of the patterned fiber’s cross-sectional structure (left) and optical image of fiber’s top view (right, scale bar, 400 μm). **b** SEM image of the patterned fiber used for TENG. **c** Enlarged image of surface pattern. **d** Schematic of TENG’s performance measurement. **e** Open-circuit voltage of both flat and patterned fiber-based TENGs. **f** Short-circuit current of both flat and patterned fiber-based TENGs. **g** Durability test for patterned fiber-based TENG. **h** Self-powered wearable multipoint touch sensor wrapped on a wrist (left) and the developed view of the touch sensor (right). Letter “A” to “G” denoting the 7 fibers respectively, while the number representing the touching sequence labeled in **i**. **i** Measured output signals from all the 7 fibers in the touch sensor.

Another feature of the DITD process is that it only modifies fiber’s surface structure without influencing its inner structure. This makes it compatible with fibers owning different complex inner structures, predicting a bright future of multifunctional fibers^50^. TENG is an energy harvesting device that converts mechanical energy including sound, vibration, tide, and wind from surroundings to electricity. It has been widely studied for powering portable devices and self-powered sensing^51,52^. Previous works^53,54^ have confirmed that introducing surface pattern can effectively enhance the output performance of TENG. Here, we assembled TENG using surface patterned fiber fabricated by the DITD technique. Figure 4a schematics the fiber’s cross-sectional structure in fiber’s axial direction (left) and displays the optical image of fiber’s top view (right, scale bar, 400 μm). PVDF was selected as cladding material because of its high electronegativity^55^, while carbon filled polyethylene (CPE) served as the electrode. Both sides of the fiber were patterned with microcylinder array as the SEM images show in Fig. 4b, c. For comparison, the flat-surface fiber with the same dimension was fabricated by the traditional fiber drawing process. Both flat and patterned fibers were cut into 10-cm long pieces. As sketched in Fig. 4d, fiber-based TENG was assembled from 5 pieces of each type of fibers that were connected in parallel (see Methods for more detail). The working mechanism of the single-electrode TENG is discussed in Supplementary Note 3, which indicates that the charge amount on the fiber surface is crucial to the performance of TENG. Creating surface pattern has been proved to be an effective approach to increase the surface charge amount as the surface pattern could increase the surface charge density and enhance the friction^53,54^. Therefore, the TENG based on surface patterned fibers is expected to offer a better performance than that based on flat fibers. Output performance of fiber-based TENG was measured, as plotted in Fig. 4e, f. The open circuit peak voltage of TENG increases from 2.1 V to 4.8 V due to the presence of surface pattern, while the short-circuit peak current of TENG increases from 160 nA to 500 nA, corresponding to the current density of 0.9 mA m^−2^ and 2.9 mA m^−2^, respectively. Moreover, a long-term stability test of TENG based on patterned fibers was conducted as shown in Fig. 4g. The TENG experienced a charge accumulation period at first and then worked stably for more than 40k working cycles. These experimental results indicate that the patterned fiber fabricated by DITD is effective and durable.

### Wearable self-powered multipoint touch sensor

A TENG with higher performance will lead to higher sensitivity and higher signal-to-noise ratio when applied to self-powered sensing applications. Therefore, based on the patterned fibers, we further developed a wearable self-powered multipoint touch sensor. As demonstrated in Fig. 4h, three 10-cm-long patterned fibers and four 8-cm-long patterned fibers were weaved into a piece of cloth and intersected at 12 sensing nodes wrapped around the wrist. When an external object such as fingers, gloves, and metal blocks approaches a certain sensing node, the charge distribution on the electrodes of the two crossed fibers connecting to this node changes because of the electrostatic induction, resulting in voltage output. Hence, the touched point can be electrically illustrated by measuring the output of the 7 fibers. The testing circuit is drawn in Supplementary Fig. 10. Both single-point and multipoint tests were performed by slight finger touches, and the output signals were recorded in Fig. 4i. The numbers labeled on the nodes in the developed view of the wearable sensor (Fig. 4h, right) represent the touching sequence under test. For instance, the first touch occurred on the node intersected by fiber B and E, matching with the result that output voltage was only detected on fiber B and E. Similarly, in the fifth touching test, output voltage was detected on fiber A, B, C, and D simultaneously, indicating a multipoint touching on the leftmost 3 nodes. All the nodes were tested during the 6 touches and the results agreed with touch behaviors very well. Additionally, all the generated voltage from the touch sensor was several volts, which is convenient to be detected.

## Discussion

In this work, we successfully developed the DITD technique for one-step and large-scale fabrication of micro/nano-patterned fibers. The surface patterns can be implemented in all directions and possess a feature size of tens of nanometers. This technique is compatible with almost all the materials and inner structures used in the thermal fiber drawing process. The thermal behavior of fiber during the DITD process was comprehensively studied via simulation and verified by experimental results. The pattern resolution, depth control, and repeatability were further examined to demonstrate the great potential of the DITD technique in a variety of applications. We investigated the plasmonic behavior of nanopatterned fiber and demonstrated a patterned fiber-based TENG device. The patterned fiber-based TENG was proved to have a higher performance than TENG fabricated using flat-surface fiber. In addition, the construction and testing of the wearable self-powered multipoint touch sensor represents the bright future of the DITD technology in multifunctional fiber-based devices, wearable electronics and smart textiles.

## Methods

### Materials

PC, PEI, PVDF, and PEEK preforms were purchased from McMaster-Carr. CPE film was purchased from GoodFellow. SEBS pellets (G1643) were purchased from Kraton. PVDF, CPE, and SEBS were used as purchased, while PC, PEI, and PEEK were prebaked at 105 °C under vacuum for 2 weeks before use.

### Templates preparation

Template used for fabricating fibers in Fiber 1b is PET-based 2D diffraction grating purchased from Yuanqiu Technology Co., Ltd., China. For fabricating fibers in Fig. 1f–h, j, a Chrome mask used in lithography was manufactured with the corresponding patterns. Then the mask was covered by a piece of PEI film and heated to 240 °C under vacuum for 30 mins. The inverted patterns were thus transferred to PEI film after cooling down and peeled off. The patterned PEI film was cut and attached on rollers to serve as the template. Templates with nanostructures corresponding to Fig. 3a, d–f were directly written under FIB (ZEISS Crossbeam 540). To prepare templates with the same pattern but different depth (Fig. 3c), a set of wafers with a groove depth of 2 μm, 8 μm, and 20 μm were fabricated beforehand via lithography and inductively coupled plasma (ICP) etching process. The similar pattern transfer process was performed to obtain surface patterned PEI films served as templates. For the template of fibers in Fig. 4a, multiple pattern transfer processes were conducted. A wafer with micro holes was firstly fabricated and a PEI film with microcylinders array was subsequently achieved after pattern transfer. Afterward, a layer of poly(dimethylsiloxane) (PDMS, Sylgard 184) was spin-coated on PEI film and cured under 100 °C for 5 min. After peeling PDMS off from PEI film, PDMS template with micro holes was completed and attached on roller surfaces for fiber fabrication.

### Fabrication of patterned fibers (DITD process)

The DITD process was performed on a fiber drawing tower built by SG Controls Ltd. An unpatterned preform was fixed on top of the tower and fed into a double heating zone furnace at a speed of 1 mm/min. The upper heating zone was used for preheating, while the lower zone was set at a higher temperature to soften the perform. The softened preform in the lower zone was continuously drawn down at a speed of 1 m/min. A neck-down region was formed in the lower zone because of fast drawing and slow feeding. A pair of rollers were fixed on a pair of 3-axis stages (Thorlabs) right below the neck-down region. The stages were adjusted to make sure the fiber was drawn down and pressed between rollers. Surface pattern was created after the fiber passing through rollers. The drawn fiber was finally collected on a capstan with a length up to kilometers long. The temperature in the upper zone was set to 200 °C, while in the lower zone, 380 °C, 380 °C, 400 °C, 500 °C, and 550 °C were set for PVDF, SEBS, PC, PEI, and PEEK, respectively. The roller is 3 cm in outer diameter and 1 cm in width with an M6 thread connected to the roller shaft, which is convenient to be fixed on an optical post. And the pressure between the rollers and fiber is kept at around 5.7 MPa for fabricating all the demonstrated patterns except for the pattern with a depth of 20 μm shown in Fig. 3c. The pattern with a depth of 20 μm depth in Fig. 3c is imprinted at a pressure of ~12 MPa.

### Fabrication of fibers used for plasmonic application

A solid PVDF preform with the width, length, and thickness of 30 mm, 200 mm, and 12 mm, respectively, is used for the DITD process. The parameters used in DITD process is provided in the above section. The resultant fiber has a width and thickness of ~1 mm and 400 μm, respectively. The drawn fiber is then cut into segments with a length of 3 cm followed by a 30-nm-thick gold film deposition process on fiber surface via electron beam evaporation.

### Preform preparation and device assembly for fiber-based TENG

The preparation of PVDF-cladding/CPE-core preform started from engraving a rectangular hole in the center of the PVDF bar’s surface. The width, length, and thickness of the PVDF bar was 30 mm, 200 mm, and 6 mm, respectively, while it was 16 mm, 120 mm, and 2 mm, respectively for the rectangular hole. CPE film was then cut and filled into the rectangular hole layer by layer. A PVDF bar with the width, length, and thickness of 30 mm, 200 mm, and 4 mm was put on CPE filled PVDF bar and the whole structure was subsequently wrapped by thread seal tape. The preform preparation finished after consolidating the wrapped preform in vacuum over under 175 °C for 2 h. The drawing temperature, speed, and imprint pressure used in the subsequent DITD process are described in the “Fabrication of patterned fibers” section. The width and thickness of the resulting fibers are around 1 mm and 400 μm, respectively.

Patterned fiber was fabricated from the preform prepared above via DITD process, while flat fiber was fabricated from the same preform via traditional fiber drawing process. Both patterned fiber and flat fiber followed the same procedure to be assembled into fiber-based TENG. The fiber was cut into short fibers with a length of 10 cm. One end of copper wire with the diameter of 50 μm was wrapped around one end of short fiber. Silver paste was then applied to connect copper wire and CPE exposed at the cross-section and dried under room temperature. A thin layer of PDMS was subsequently painted onto the surface of dried silver paste to encapsulate the connection area for a stable electrical contact. The assembly of fiber-based TENG ended by connecting five copper wires from five short fibers together.

### Characterizations

The micro/nano surface pattern was observed under field emission SEM (JEOL 7600 F). The optical images for fiber’s cross-sections and surface topography were captured by microscopy (Olympus BX51). Thermal images were taken by thermal camera (FLIR T540). The dark-field scattering spectra measurement was conducted by Cytoviva hyperspectral imaging system. Both patterned fiber-based TENG and flat fiber-based TENG were tested under the same condition. The TENGs were driven by a linear motor (LinMot E1100). The open-circuit voltage was measured by an electrometer (Keithley 6517B), while the short-circuit current was measured by a preamplifier (Stanford Research Systems SR570). Both voltage signal and current signal were collected by an analog input module (NI-9125) inserted in NI CompactDAQ Chassis (cDAQ-9171) which was connected to computer.

## Supplementary information

Supplementary Information

Description of Additional Supplementary Information

Supplementary Movie 1

## Data Availability

The data that support the findings of this study are available from the corresponding author upon reasonable request.
