# Supplementary material for: Designer patterned functional fibers via direct imprinting in thermal drawing
Source: Nat Commun. 2020 Jul 31;11:3842. doi: 10.1038/s41467-020-17674-8 (PMC7395721; doi:10.1038/s41467-020-17674-8)
Supplement: Supplementary file 1 — Supplementary Information [file 41467_2020_17674_MOESM1_ESM.pdf]

Supplementary Information for

Designer Patterned Functional Fibers via Direct Imprinting in Thermal Drawing

Wang et al.

Supplementary Note 1. Polymer flow behavior during imprint process

The imprint process in our work could be considered as pressing the raised part of the template (indenter) into the polymer fiber under external pressure (pressed by rollers), while the polymer fills into the concave part of the template (cavity). Thus, understanding the flow behavior during the imprint process is crucial towards high-fidelity pattern transfer, i.e., creating high-resolution surface patterns on fibers.

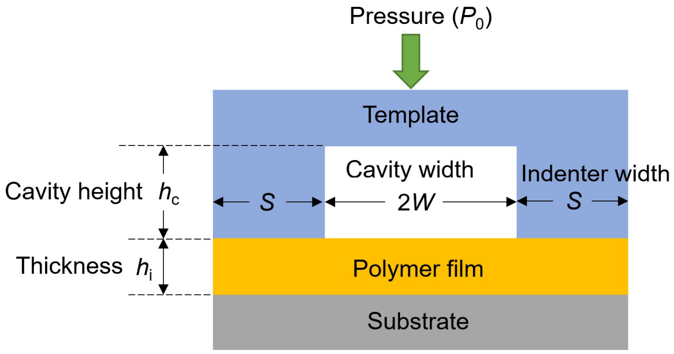

Supplementary Figure 1. A simplified model for the imprint process.

As sketched in Supplementary Fig. 1, our discussion on the flow behavior will base on a simplified model, where a template with a rectangular cavity is pressed to a polymer film on a planar hard substrate. This rectangular cavity is the unit template structure of both our striped pattern and many other works in nanoimprint lithography[1-3].

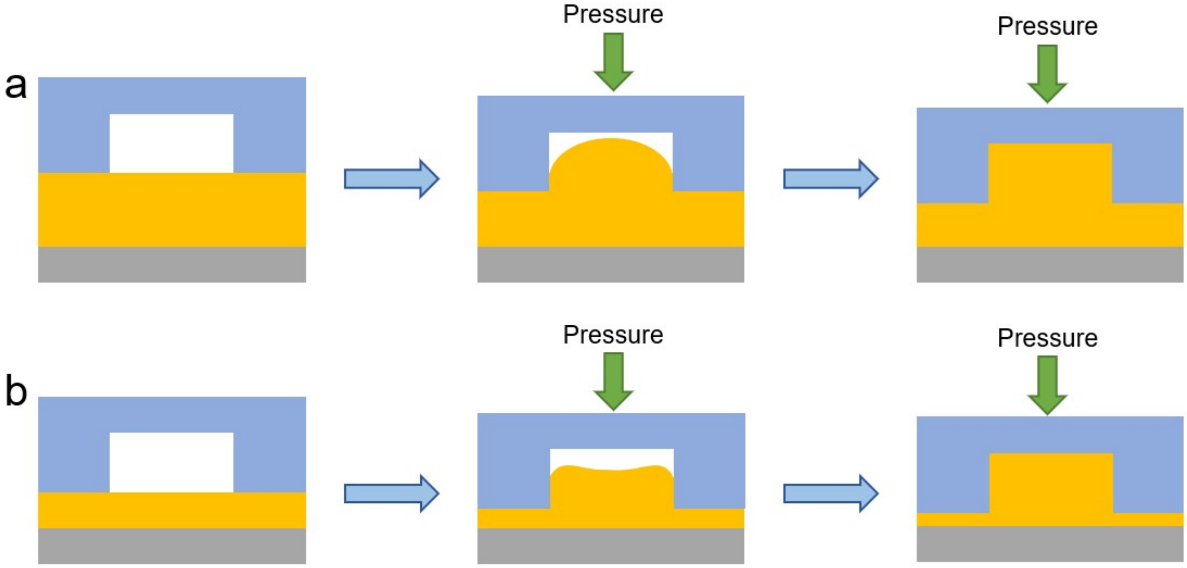

Supplementary Figure 2. Two flow modes when the polymer fills into the cavity. (a) Single peak formed. (b) Double peaks formed

There are two possible flow modes as the indenter is pressed downward and the polymer flows into the cavity[2, 3]. One is a single polymer peak formed in the middle of the cavity (Supplementary Fig. 2a). In the case that the polymer film is thick, the polymer near the sidewall will flow downward driven by the downward shearing force from the sidewall, which has less influence on the polymer in the middle of the cavity. Thus, a peak in the middle is formed. The other flow mode

is double polymer peaks formed near the cavity sidewalls corresponding to Supplementary Fig. 2b. This mode occurs when the polymer film is not thick enough. The polymer between the indenter and substrate will form a recognizable literal flow towards the cavity because of the limited space. This lateral flow will slow down after entering the cavity as it is driven by the shear near the indenter. Hence, a larger amount of polymer will stay near the two sidewalls, forming the double polymer peaks.

H. Rowland et al. put forward a criterion to predict which flow mode will occur during imprinting based on their simulation results[3]. They found that single polymer peak would occur when the ratio of cavity half-width (W) to initial film thickness (h_i) is smaller than 1.2. Otherwise, the double polymer peak will be formed. This criterion agrees well with other reported works except for small variations around the ratio value of 1.2.

Here in our Direct Imprinting in Thermal Drawing (DITD) process, The thicknesses of our drawn fibers are mostly around 360 μm. the cavity widths ($2W$) we used in our template are ranging from 300 nm to 20 μm. Considering that the fiber may be patterned on both sides, we take h_i as 180 μm. Thus, the ratios of W/h_i in our work are ranging from 8.3E-4 to 0.06, which is much smaller than 1.2. Thus, we could predict that a single polymer peak will occur in our imprint process (Supplementary Fig. 2a). In fact, the thickness of the fiber produced via the thermal drawing process is generally larger than 100 μm. Therefore, the flow mode will always be single peak mode in the DITD process except for producing micro/nanopatterned fibers with the pattern size larger than the fiber thickness, which is hundreds of micrometers.

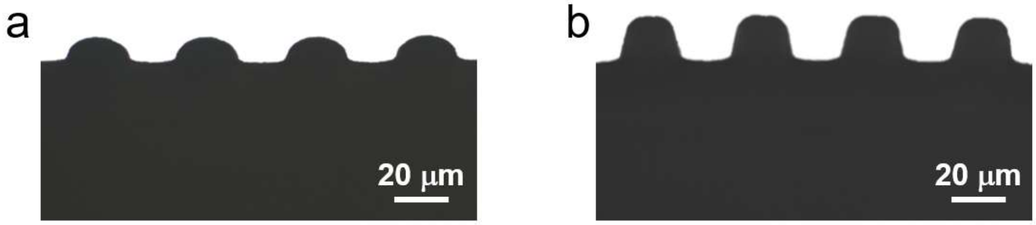

Supplementary Figure 3. Experimental verification of the single peak flow mode. (a) Patterned fiber drawn under low imprint pressure (~6 MPa), showing the middle state of the polymer filling into the cavity. (b) Patterned fiber drawn under high imprint pressure (~12 MPa).

Further experiments are conducted to verify that this model and criterion is applicable to our imprint process. A template with a square cavity as the unit structure is used. And both the cavity width ($2W$) and cavity height (h_c) are 20 μm. Two kinds of fibers are drawn under the same condition and imprinted by the same template except for different applied pressure as shown in Supplementary Fig. 3. As the lower pressure will lead to a longer time for the polymer to fully fill the cavity, the middle state of polymer flow in our imprint process could thus be observed as the contact time between roller and fiber is shorter than the needed time to fully fill the polymer. A single peak mode is exhibited in Supplementary Fig. 3a, which agrees well with the analysis above.

Besides cavity half-width to initial film thickness ratio (W/h_i), the polymer supply ratio (S/h_r) is also important factors that influence the polymer flow behavior[3]. Polymer supply ratio is defined as the ratio of indenter width (S) to residual film thickness (h_r), which can be obtained as $h_r =$

$h_i - \frac{Wh_c}{S+W}$. From this expression, we can find that a larger cavity height (h_c) or thinner film (smaller h_i) will lead to a smaller residual film thickness (h_r). A small h_r indicates that the indenter will move very close to the hard substrate as the polymer flows into the cavity, which may lead to squeeze flow. And a wide indenter (large *S*) will also promote the squeeze flow. The squeeze flow will become important in polymer flow behavior when $S/h_r > 1$. Similarly, we will estimate the polymer ratio in our work. The largest indenter width (*S*) we use is 10 μm, while the largest cavity height h_c is also 20 μm. And the $W/(W+S)$ we use is around 0.5. Taking the same h_i = 180 μm as we estimated above, we can calculate that the largest polymer supply ratio is around 0.06, much less than 1. This small ratio indicating that the squeeze is negligible in our imprint process. Additionally, to reach a large polymer supply ratio, the cavity volume should be large enough to be comparable with the initial film volume, which could be a less common configuration in micro/nanoscale surface patterns as our drawn fiber is commonly larger than 100 μm. Therefore, squeeze flow is commonly negligible in the DITD process except for some special cases where both *S*, *W*, and h_c are designed in hundreds of micrometers and meanwhile a small fiber thickness (h_i) is required.

To decide the flow pattern (laminar flow or turbulence flow), Reynolds number ($Re = \rho u D/\eta$) should be examined. In the expression, ρ is the density of the polymer, u is the flow speed, D is the width of the cavity ($2W$) in our case, and η is the viscosity of the polymer. In our DITD process, we should always use a polymer that is compatible with the thermal drawing process. Thus, the viscosity range is 10^4 Pa·s to 10^8 Pa·s. For micro/nanopatterns, $2W$ is ranging from 10^{-8} m to 10^{-4} m. And polymer density could be estimated at the level of 10^3 Kg/m^3. Thus, the value of $\rho D/\eta$ should be ranging from 10^{-5} s/m to 10^{-14} s/m, indicating a very small Reynolds number. Thus, the flow pattern in our imprint process is laminar flow.

Based on the estimation above, we can infer the polymer flow behavior: the polymer will flow into the cavity in a laminar pattern and form a single peak in the middle of the cavity and no obvious literal flow or squeeze flow could be observed. Thus, we can estimate the flow speed when filling the cavity based on this behavior. We assume that the polymer is incompressible, and the laminar flow is steady and in parallel with the sidewalls of the cavity. Thus, it follows the continuity equation $\partial u/\partial x = 0$ (x is the direction is perpendicular to the sidewalls.). Applying the boundary conditions as we assumed above to the Navier–Stokes equations in fluid mechanics[4], the character flow velocity (*V*) can be obtained as follows.

$$V = D^2P/3\eta L \tag{1}$$

where *D* is the half cavity width (*W*), L is the character distance, and P is the pressure difference between the character distance. In this model, we take L as cavity height (h_c), and *P* as the pressure imposed on the polymer through the indenter ($P = P_0\frac{W+S}{S}$). Thus, the character flow velocity can be expressed as follows.

$$V = P_0(2W)^2(S+W)/12\eta h_c S \tag{2}$$

Also, the character fill time for polymer filling can be estimated by taking h_c as the character distance.

$$t = h_c/V = 12\eta S h_c^2 / P_0 (2W)^2 (S + W) \tag{3}$$

Here, we take the cavity size from a template used in our work for example. The S, W, and h_c are $2.5*10^{-6}$ m, $2.5*10^{-6}$ m, and $1*10^{-7}$ m, respectively. P is estimated to be 5.7 MPa as discussed in Supplementary Note 2, while a typical viscosity of 10^6 Pa·s is used. And thus, the character flows velocity and fill time can be estimated to be $2.38*10^{-4}$ m/s and 0.42 ms, respectively.

Based on the flow velocity, we can also estimate the capillary number (Ca), which is a dimensionless quantity in fluid dynamics to evaluate whether the flow is dominated by viscous drag force or surface tension. Ca is defined as $\eta V/\gamma$, where η is the viscosity, V is the velocity, and γ is the surface tension. A high capillary number (Ca > 1) represents that the flow is dominated by viscous force, while a low capillary number (Ca < 1) indicating a surface tension force dominating the flow. Using the parameters above and taking the surface tension of the polymer as 0.02 N/m[5], Ca is estimated to be $1.2*10^4$, indicating that the surface tension force is very weak in this case. Generally, in the DITD process, the polymer we used must be compatible with the thermal drawing process, which determines a high viscosity between 10^4 Pa·s to 10^8 Pa·s. And the surface tensions of the polymers are usually at the level of 10^{-2} N/m. Therefore, the Ca is expected to be a very large value in the DITD process except in some special cases where the flow velocity is very small and meanwhile the viscosity is quite low.

It is worth nothing to mention that for an independent nanoimprinting process, regardless of the flow behavior, the cavity-filling under pressure could always be completed as long as the time is long enough. However, in the DITD process, the imprint process is combined with the thermal drawing process, leading to a limited imprint time, i.e., the contact time between fiber and patterned rollers, as the fibers are kept drawing. If the character fill time of a certain imprint process is longer than the contact time, the polymer will not be able to fully fill the cavity in the template, resulting in a heavily distorted pattern, like the case shown in Supplementary Fig. 3a. Hence, special attention should be paid to the factors influencing the fill time when designing a DITD process. To be more specific, we divide the expression of fill time into three terms as follows.

$$t = 12 * \eta * \left(\frac{h_c}{2W}\right)^2 * \frac{1}{P} \tag{4}$$

The term $h_c/2W$ represents the pattern aspect ratio (cavity height to width). η is the material property that largely depends on the temperature. And the P here denotes the pressure applied to the polymer, which is $P_0 * \frac{W+S}{S}$ in the model we discussed. Thus, for a given fiber material, the fill time is mainly determined by three factors: temperature, pattern aspect ratio, and applied pressure. To achieve a high-resolution surface patterned fiber via DITD process, these three factors should be properly designed to match with the contact time. The contact time is mainly decided by the fiber drawing process, which will be discussed in Supplementary Note 2.

Supplementary Note 2. Estimation of contact time and contact pressure between rollers and fiber.

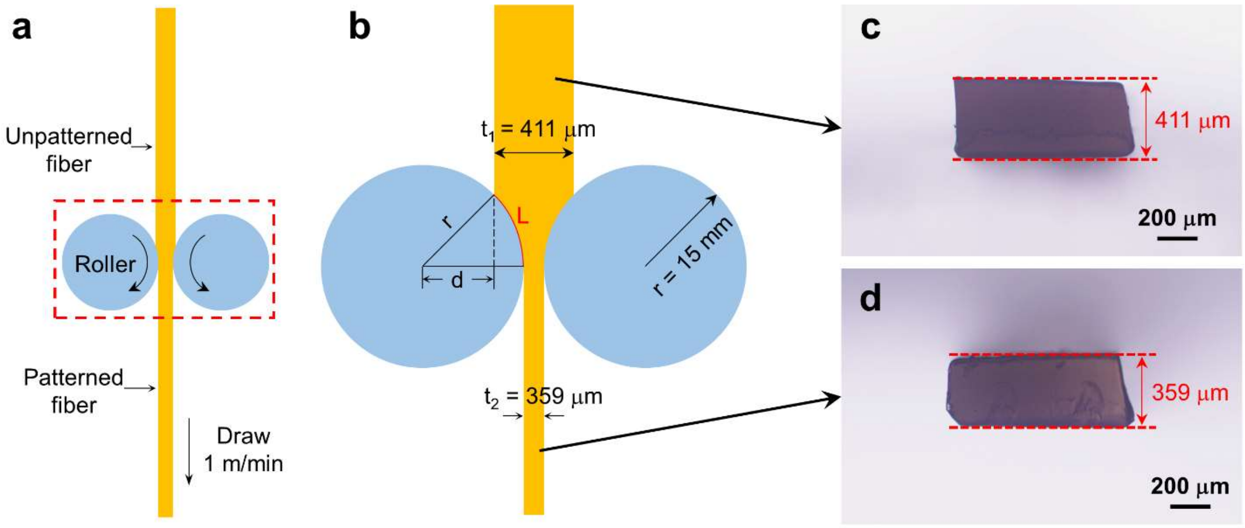

Supplementary Figure 4. **Estimation of contact length between rollers and fiber.** (a) Model for estimating contact length. (b) Enlarged sketch showing the contact area and fiber deformation. (c) Cross-section image of fiber before patterned. (d) Cross-section image of fiber after patterned.

We assume that the rollers were placed symmetrically on both sides of the fiber. As sketched in Supplementary Fig. 4a, the patterned fiber will be slightly thinner than the unpatterned fiber after pressed by rollers during the imprint process. To see it clearer, the contact area and fiber deformation were exaggerated in Supplementary Fig. 4b. The thickness of the fiber before and after imprint was measured under a microscope as shown in Supplementary Fig. 4c and d. Then we can estimate the contact length (L) according to the actual sizes of fiber and rollers:

$$L = r * \cos^{-1}\frac{d}{r} = r * \cos^{-1}\frac{r - \frac{t_1 - t_2}{2}}{r} = 0.88\, mm \quad (5)$$

Note that the drawing speed (v) is 1 m/min, the contact time should be $t = L/v = 53$ ms. The width (w) of the patterned fiber is around 1 mm while the force (F) we use for imprint is around 5 N. Thus, the contact pressure can be estimated to be $P = {}^{F}/_{w * l} = 5.68\, MPa$.

Supplementary Note 3. Working mechanism of single-electrode triboelectric nanogenerator (TENG).

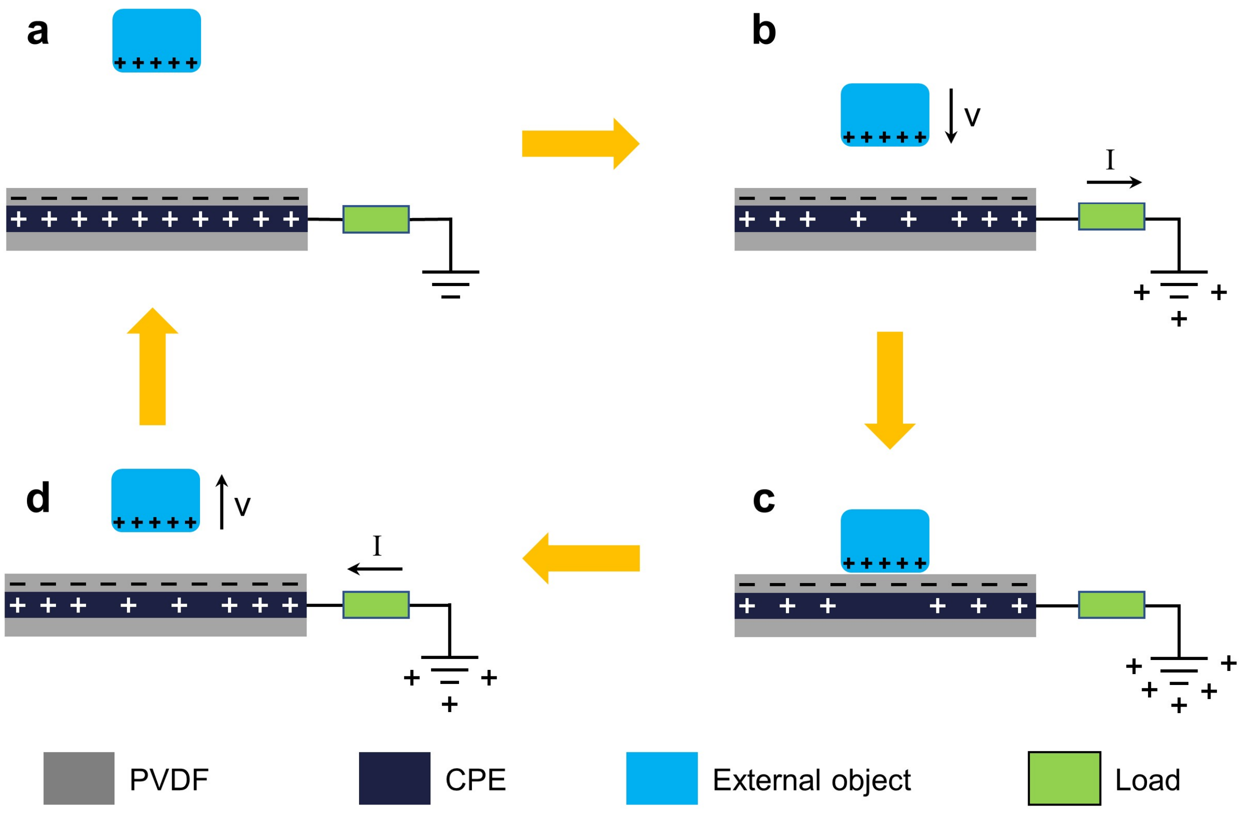

Supplementary Figure 5. **Working mechanism of single electrode TENG.** (a) External object is far away from the PVDF fiber. (b) External object is moving to the PVDF fiber. (c) External object contacts with the PVDF fiber. (d) External object is moving away from the PVDF fiber.

When external object friction with Polyvinylidene fluoride (PVDF), the electron on the surface of the external object will be transferred to the surface of PVDF because of its strong electronegativity. Thus, the surface of PVDF will bear negative charge while the external object bears positive charge. Positive charge will be generated on carbon filled polyethylene (CPE) electrode inside PVDF fiber because of electrostatic induction, as shown in Supplementary Fig. 5a. As the positive charge on the object is moving close to the PVDF fiber, the electrostatic potential of the electrode will increase, resulting in the positive charge in the electrode flow towards the ground through the load (Supplementary Fig. 5b). When the object contacts the PVDF fiber, a large amount of positive charge will move to the ground as sketched in Supplementary Fig. 5c. Similarly, when the object moves away from the PVDF fiber, the positive charge will flow back to the electrode as the potential of electrode decreases, generating current from ground to the electrode (Supplementary Fig. 5d). Finally, the charge distribution will go back to the original state after the object moves far away from the PVDF fiber. As the external moves close to and away from the PVDF fiber repeatedly, an alternating current will flow through the load. That is, mechanical energy is transformed into electricity.

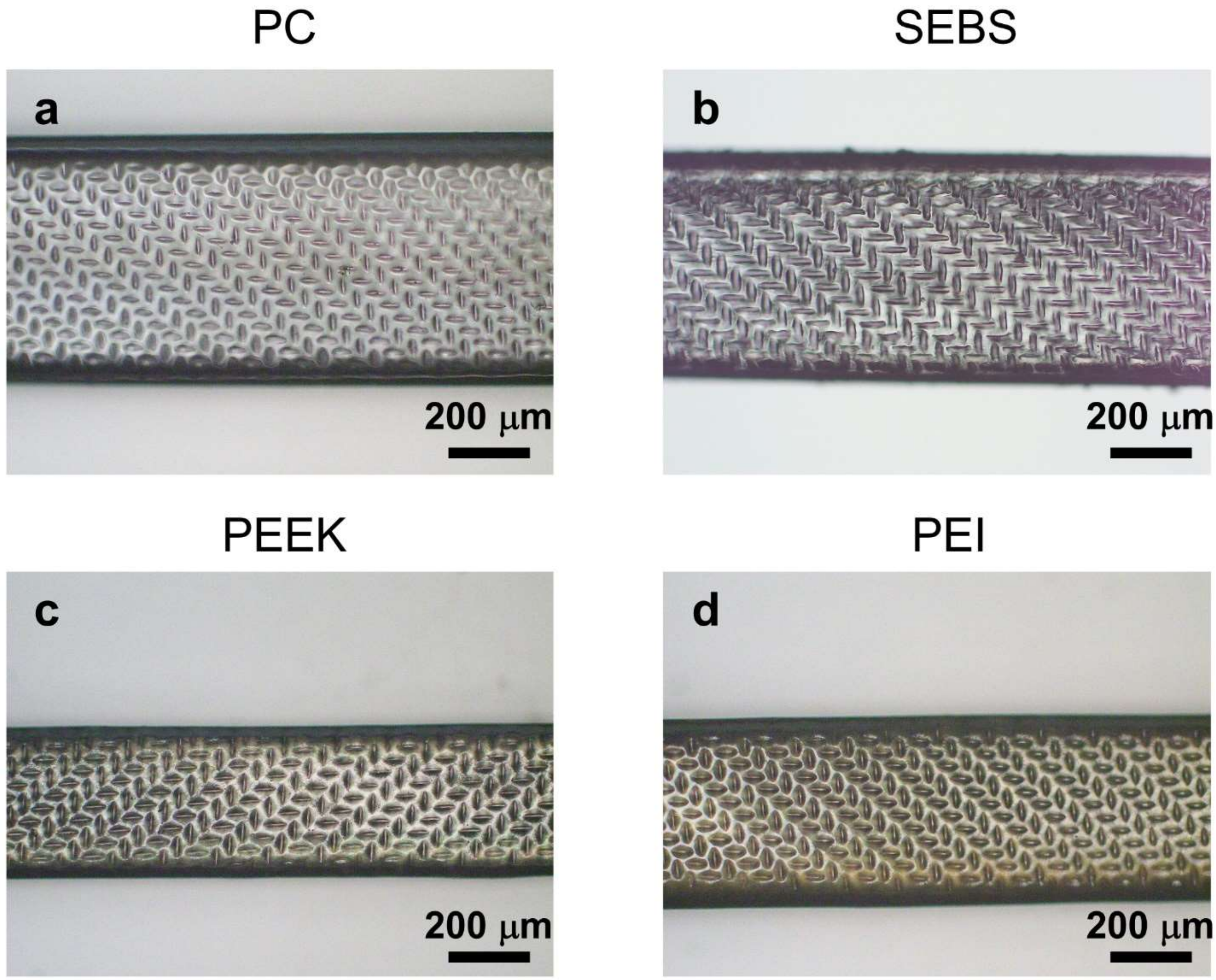

Supplementary Figure 6. **Surface pattern created on fibers with different materials.** (a) Polycarbonate (PC) fiber. (b) Styrene-ethylene-butylene-styrene (SEBS) fiber. (c) Polyetheretherketone (PEEK) fiber. (d) Polyetherimide (PEI) fiber.

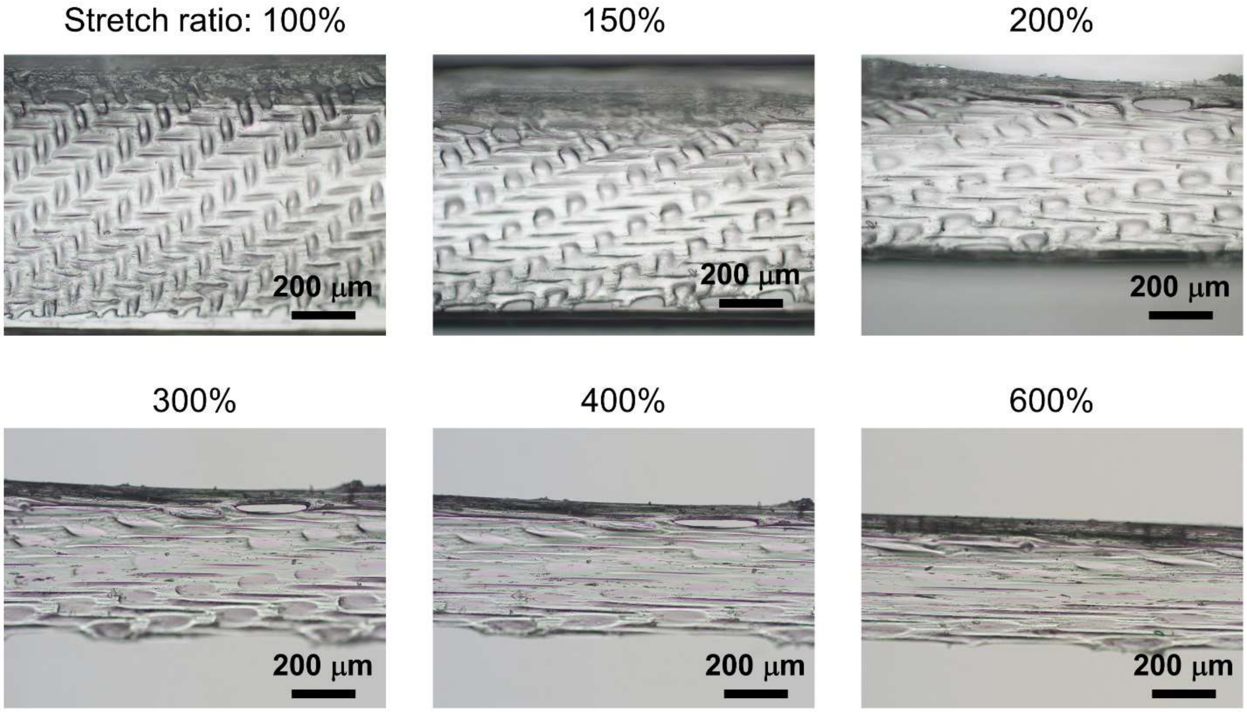

Supplementary Figure 7. **Patterned SEBS fiber under different stretch ratios.**

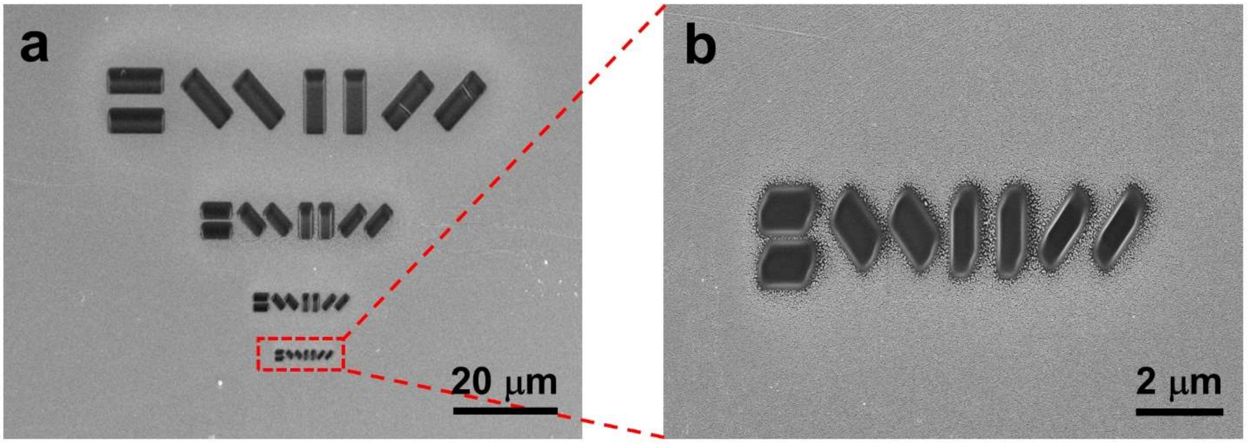

Supplementary Figure 8. Scanning electron microscope (SEM) images of the template used for creating the resolution test pattern. (a) Overview of resolution test pattern. (b) Enlarged image for the smallest pattern

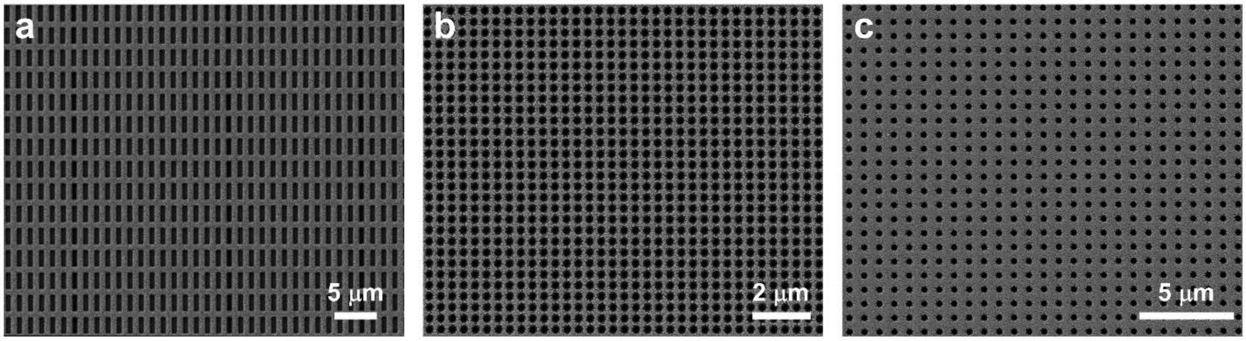

Supplementary Figure 9. SEM images of the nanopatterned templates. (a) Rectangular holes with a length of 2.1 μm and a width in 700 nm in width. (b) Circular holes with a radius of 150 nm and a period of ~ 400 nm. (c) Circular holes with a radius of 150 nm and a period of ~ 800 nm.

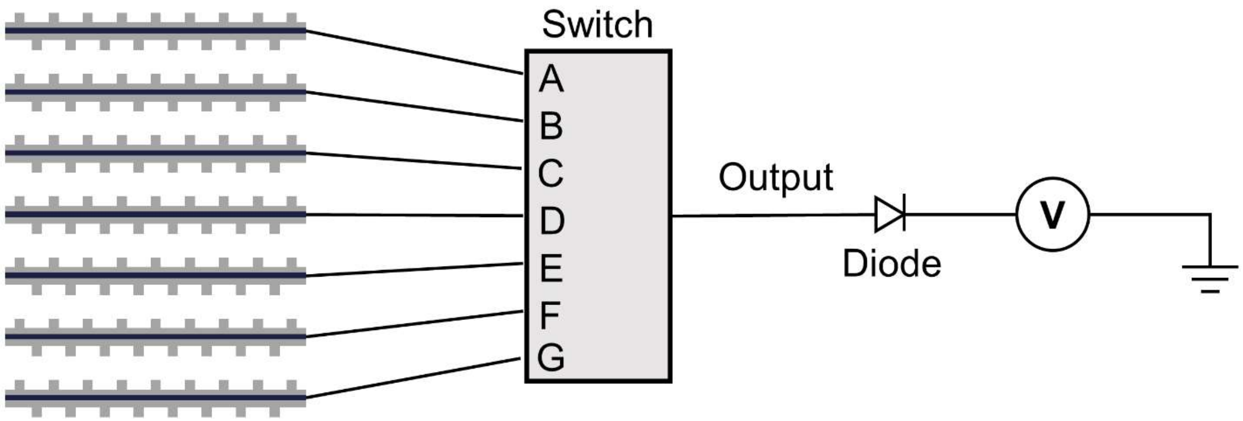

Supplementary Figure 10. Testing circuit for the self-powered wearable multipoint touch sensor.

Supplementary References:

1 Cross, G. L. W., Langford, R. M., O'Connell, B. S. & Pethica, J. B. The mechanics of nanoimprint forming. *MRS Proceedings* **841,** (2011).

2 Dumond, J. J. & Low, H. Y. Recent developments and design challenges in continuous roller micro- and nanoimprinting. *J. Vac. Sci. Technol. B* **30,** 010801 (2012).

3 Rowland, H. D., Sun, A. C., Schunk, P. R. & King, W. P. Impact of polymer film thickness and cavity size on polymer flow during embossing: Toward process design rules for nanoimprint lithography. *J. Micromech. Microeng.* **15,** 2414-2425 (2005).

4 Munson, B. R., Young, D. F., & Okiishi, T. H. *Fundamentals of fluid mechanics. pp. 322–323* (Wiley, New York, 2013).

5 Nguyen-Dang, T. et al. Controlled sub-micrometer hierarchical textures engineered in polymeric fibers and microchannels via thermal drawing. *Adv. Funct. Mater.* **27,** 1605935 (2017).
