# Supplementary material for: Designer patterned functional fibers via direct imprinting in thermal drawing
Source: Nat Commun. 2020 Jul 31;11:3842. doi: 10.1038/s41467-020-17674-8 (PMC7395721; doi:10.1038/s41467-020-17674-8)
Supplement: Supplementary file 2 — Description of Additional Supplementary Information [file 41467_2020_17674_MOESM2_ESM.pdf]

Description of Additional Supplementary Files

File Name: Supplementary Movie 1
Description: Continuous fabrication of fiber with surface pattern.
